# Performance metrics for models designed to predict treatment effect

**DOI:** 10.1186/s12874-023-01974-w

**Published:** 2023-07-08

**Authors:** C. C. H. M. Maas, D. M. Kent, M. C. Hughes, R. Dekker, H. F. Lingsma, D. van Klaveren

**Affiliations:** 1 Department of Public Health, Erasmus University Medical Center, Doctor Molewaterplein 40, 3015 GD Rotterdam, Netherlands; 2 Predictive Analytics and Comparative Effectiveness Center, Institute for Clinical Research and Health Policy Studies, Tufts Medical Center, Boston, USA; 3 Erasmus School of Economics, Erasmus University Rotterdam, Rotterdam, Netherlands

**Keywords:** Heterogeneous treatment effect, Prediction models, Logistic regression, Causal forest

## Abstract

**Background:**

Measuring the performance of models that predict individualized treatment effect is challenging because the outcomes of two alternative treatments are inherently unobservable in one patient. The C-for-benefit was proposed to measure discriminative ability. However, measures of calibration and overall performance are still lacking. We aimed to propose metrics of calibration and overall performance for models predicting treatment effect in randomized clinical trials (RCTs).

**Methods:**

Similar to the previously proposed C-for-benefit, we defined observed pairwise treatment effect as the difference between outcomes in pairs of matched patients with different treatment assignment. We match each untreated patient with the nearest treated patient based on the Mahalanobis distance between patient characteristics. Then, we define the E_avg_-for-benefit, E_50_-for-benefit, and E_90_-for-benefit as the average, median, and 90^th^ quantile of the absolute distance between the predicted pairwise treatment effects and local-regression-smoothed observed pairwise treatment effects. Furthermore, we define the cross-entropy-for-benefit and Brier-for-benefit as the logarithmic and average squared distance between predicted and observed pairwise treatment effects. In a simulation study, the metric values of deliberately “perturbed models” were compared to those of the data-generating model, i.e., “optimal model”. To illustrate these performance metrics, different modeling approaches for predicting treatment effect are applied to the data of the Diabetes Prevention Program: 1) a risk modelling approach with restricted cubic splines; 2) an effect modelling approach including penalized treatment interactions; and 3) the causal forest.

**Results:**

As desired, performance metric values of “perturbed models” were consistently worse than those of the “optimal model” (E_avg_-for-benefit ≥ 0.043 versus 0.002, E_50_-for-benefit ≥ 0.032 versus 0.001, E_90_-for-benefit ≥ 0.084 versus 0.004, cross-entropy-for-benefit ≥ 0.765 versus 0.750, Brier-for-benefit ≥ 0.220 versus 0.218). Calibration, discriminative ability, and overall performance of three different models were similar in the case study. The proposed metrics were implemented in a publicly available R-package “HTEPredictionMetrics”.

**Conclusion:**

The proposed metrics are useful to assess the calibration and overall performance of models predicting treatment effect in RCTs.

**Supplementary Information:**

The online version contains supplementary material available at 10.1186/s12874-023-01974-w.

## Introduction

Clinicians and patients generally select the treatment that is expected to be beneficial on average for the patient population. However, the average treatment effect (ATE) for a population does not accurately reflect the effect of treatment for each patient individually [1–3]. Various models have been proposed for predicting individualized treatment effects in a randomized clinical trial (RCT) [4–6]. These models aim to predict the difference between the outcomes of two alternative treatments for each patient.

Usually, only one of the outcomes can be observed for a given patient, the counterfactual outcome remains unobserved. This phenomenon–known as the fundamental problem of causal inference–complicates the assessment of a model’s ability to predict treatment effect [7]. As a result, the performance of models that predict treatment effect cannot be quantified with conventional metrics evaluating risk predictions [8]. To resolve this issue, observed pairwise treatment effect can be defined as the difference between outcomes in pairs of matched patients. Recently, the C-for-benefit has been proposed for quantifying to what extent the models can discriminate between patients who benefit and those who do not [9]. However, measures of calibration–the agreement between predicted and observed treatment effect in *groups* of patients–and measures of overall performance–the discrepancy between predicted and observed treatment effect across *individual* patients–are still lacking.

**Fig. 1:**
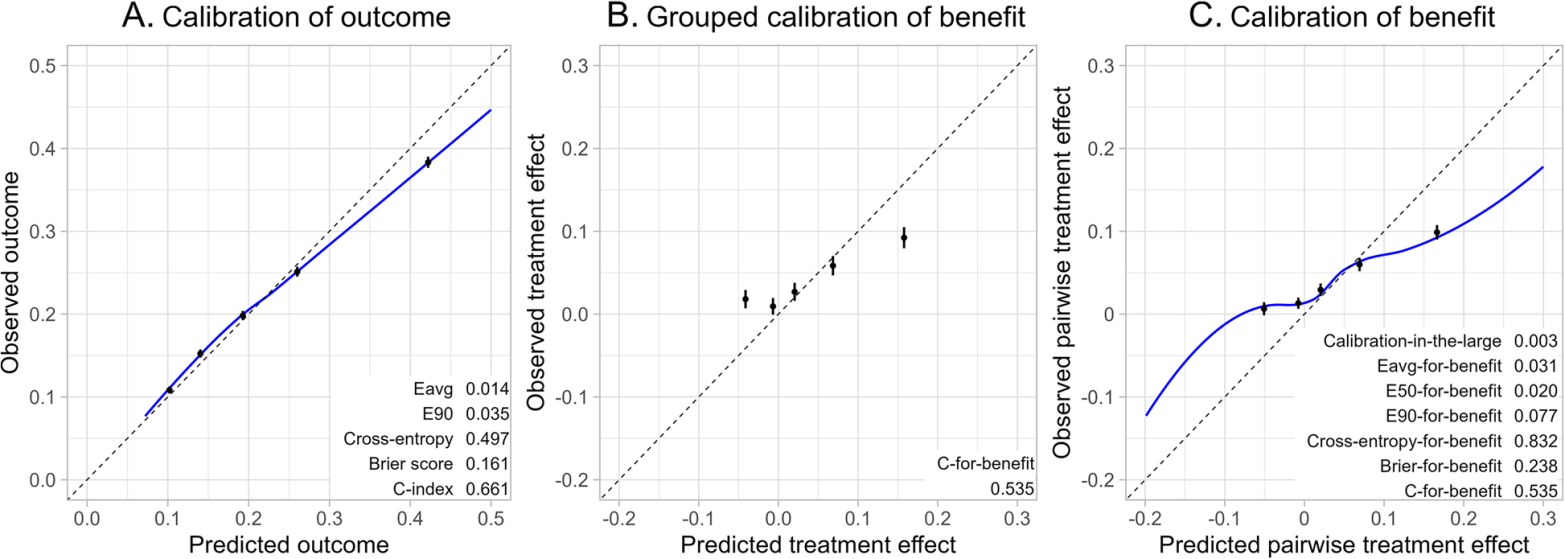
An illustration of risk and benefit calibration figures with performance metrics of simulated data. We sampled (*n* = 3,600) from a simulated trial super population (100,000) with 12 binary risk predictors with 6 true treatment interactions [13]. Panel **A** depicts observed outcome versus predicted outcome by local regression (blue line, displayed between 0 and 0.5) and quantiles of predicted outcome (black dots), with the E-statistics, cross-entropy, Brier score, and C-index. Panel **B** depicts the calibration for benefit in groups with 95% confidence intervals, with the C-for-benefit. Panel **C** depicts observed versus predicted pairwise treatment effect by local regression (blue line, displayed between -0.2 and 0.3) and quantiles of predicted pairwise treatment effect (black dots), with the newly proposed metrics

For models predicting outcome risk and not treatment effect, several metrics are available to assess calibration (i.e., E-statistic), and overall performance (i.e., cross-entropy and Brier score) [10–12]. However, these metrics may poorly reflect a model’s ability to predict treatment effect. For example, in a simulation scenario with a relatively small simulated data sample, the risk predictions of a model with all possible treatment interactions are reasonably well calibrated (Fig. 1A), while the corresponding treatment effect predictions are poorly calibrated (Fig. 1B) [13]. Apart from such graphical assessment of calibration in groups of patients with similar predicted treatment effects, no metrics are available that quantify the calibration or the overall performance of treatment effect predictions [14]. Therefore, we aimed to extend these performance metrics for calibration and overall performance for risk prediction models that are designed to predict treatment effect in RCTs.

## Methods

### Definition of treatment effect

With the potential outcomes framework, we can define the (conditional average) treatment effect $\tau (x)$ for a patient with baseline characteristics $X$ as the expected difference between ${Y}_{i}(0)$, i.e., the outcome that would have been observed under the treatment value $W=0$, and ${Y}_{i}\left(1\right)$, i.e., the outcome that would have been observed under $W=1$, conditional on the patient characteristics $X$, i.e. [15],

$$\tau \left(x\right)=E\left[{Y}_{i}\left(0\right)-{Y}_{i}\left(1\right)|{X}_{i}=x\right]$$

Here, the event associated with the outcome was assumed to be unfavorable. Thus, treatment benefit, i.e., a positive $\tau \left(x\right)$, is expected when the outcome probability under control treatment is higher than the outcome probability under active treatment. Alternatively, two active treatments can be administered instead of control and active treatment.

### Metrics based on the matching principle

Using the matching principle, we defined observed pairwise treatment effect as the difference in outcomes between two similar patients with different treatment assignments (Table 1) [9]. Similarity was based on baseline patient characteristics to create pairs of similar patients with different treatment assignments. Specifically, we matched each untreated patient with the nearest treated patient based on the Mahalanobis distance between the patient characteristics without replacement [16]. We performed a sensitivity analysis to assess how sensitive the proposed metrics are to the choice of the matching procedure (Additional file 1). With a binary outcome (say, 0 for alive and 1 for dead), four outcome combinations are possible for a pair of patients. First, treatment benefit was indicated if the treated patient lives and the untreated patient dies. Second, treatment harm was indicated if the treated patient dies and the untreated patient lives. Lastly, no effect of treatment was indicated if both the treated and untreated patients live, or if both die. Thus, the observed pairwise treatment effect takes the values 1 (benefit), 0 (no effect), and -1 (harm). Concurrently, predicted pairwise treatment effect is the difference between the predicted outcome probability of the untreated patient minus the predicted outcome probability of the treated patient. We illustrate the calculation of the proposed metrics based on a small artificial sample (Table 1). All of the following metrics using this matching principle were added to Fig. 1C for illustration. The proposed metrics were implemented in a publicly available R-package “HTEPredictionMetrics” [17].

**Table 1:** An illustration of the calculation of the proposed metrics based on matching patients to assess models predicting treatment effect

	**Patient assigned to treatment**				**Patient assigned to control treatment**				**Matched pair**				
**Matched patient pair (A)**	${{\varvec{p}}}_{0}$ **(B)**	${{\varvec{p}}}_{1}$ **(C)**	**Predicted treatment effect (D = B-C)**	**Observed outcome (E)**	${{\varvec{p}}}_{0}$ **(F)**	${{\varvec{p}}}_{1}$ **(G)**	**Predicted treatment effect (H = F-G)**	**Observed outcome (I)**	${{\varvec{p}}}_{0}$ **(J = F)**	${{\varvec{p}}}_{1}$ **(K = C)**	**Predicted pairwise treatment effect** **(L = J-K)**	**Observed pairwise treatment effect (M = E-I)**	**LOESS curve (N)**
1	0.136	0.283	-0.147	1	0.162	0.307	-0.145	1	0.162	0.283	-0.121	0	-0.412
2	0.246	0.343	-0.097	0	0.218	0.319	-0.101	1	0.218	0.343	-0.125	-1	-0.589
3	0.156	0.219	-0.063	1	0.142	0.203	-0.061	0	0.142	0.219	-0.077	1	0.901
4	0.081	0.083	0.002	0	0.098	0.062	0.036	0	0.098	0.083	0.015	0	-0.081
5	0.345	0.212	0.133	1	0.299	0.171	0.128	0	0.299	0.212	0.087	1	0.937
6	0.421	0.390	0.031	1	0.561	0.255	0.306	1	0.561	0.390	0.171	0	0.190
7	0.364	0.201	0.163	1	0.243	0.164	0.079	1	0.243	0.201	0.042	0	0.217
8	0.264	0.199	0.065	1	0.345	0.278	0.067	0	0.345	0.199	0.146	1	0.707

The calibration metrics are calculated in the following manner calibration-in-the-large = abs(mean(M)-mean(N)) $\approx$ 0.016, E_avg_-for-benefit = mean(abs(L-N)) ≈ 0.429, E_50_-for-benefit = median(abs(L-N)) ≈ 0.378, and E_90_-for-benefit = quantile(abs(L-N), 0.9) ≈ 0.888. The overall performance are calculated by Cross-entropy-for-benefit $=-\frac{1}{{n}_{p}}\left[I\left(M=1\right)\cdot \mathrm{log}\left[\left(1-K\right)J\right]+I\left(M=0\right)\mathrm{log}\left[\left(1-K\right)\left(1-J\right)+K\cdot J\right]+ I\left(M=-1\right)\mathrm{log}\left[K\left(1-J\right)\right]\right]\approx 1.001$ and Brier-for-benefit $=\frac{1}{2{n}_{p}}\left[{\left[\left(1-K\right)J-I\left(M=1\right)\right]}^{2}+{\left[\left(1-K\right)\left(1-J\right)+K\cdot J-I\left(M=0\right)\right]}^{2}+{\left[K\left(1-J\right)-I\left(M=-1\right)\right]}^{2}\right] \approx 0.308$, where n_p_ the number of patient pairs. *Abbreviations*: p_0_ = P(Y = 1│W = 0); p_1_ = P(Y = 1│W = 1); LOESS curve is created by predict(stats::loess(M ~ L))h﻿

#### Calibration

Calibration refers to the correspondence between the predicted and observed treatment effects. The calibration-in-the-large or mean calibration was defined as the average observed pairwise treatment effect minus the average predicted pairwise treatment effect [18]. If the algorithm overestimates treatment effect, the average predicted pairwise treatment effect is higher than the observed pairwise treatment effect, resulting in a negative calibration-in-the-large value. Conversely, the calibration-in-the-large will be positive if treatment effect is underestimated.

Calibration can also be assessed by a smoothed calibration curve obtained by a local regression of observed pairwise treatment effect on predicted pairwise treatment effect, with default values for the span and the degree of polynomials (Fig. 1C). Similar to the E-statistic and the Integrated Calibration Index, we propose to measure calibration by the average absolute vertical distance between this smoothed calibration curve and the diagonal line of perfect calibration [10]. This quantity, which we named the E_avg_-for-benefit, can be interpreted as the weighted difference between observed pairwise treatment effect and predicted pairwise treatment effect, with weights determined by the empirical density function of the predicted pairwise treatment effect. Similarly, we defined the E_50_-for-benefit and the E_90_-for-benefit as the median and 90^th^ percentile of the absolute differences between the predicted pairwise treatment effect and the smoothed observed pairwise treatment effect (Table 1) [10]. Thus, the E-statistics indicate perfect calibration when zero.

#### Discrimination

Discrimination refers to a model’s ability to separate patients who benefit from treatment and those who do not. To measure discrimination, we used the previously proposed C-for-benefit, i.e., the probability that from two randomly chosen matched patient pairs with unequal observed pairwise treatment effect, the pair with greater observed pairwise treatment effect also has a larger predicted pairwise treatment effect [9]. The C-for-benefit was calculated by the number of concordant pairs divided by the number of concordant and discordant pairs. Two patient pairs are concordant if the pair with the larger observed pairwise treatment effect also has a larger predicted pairwise treatment effect. Two patient pairs are discordant if the pair with larger observed benefit has a smaller predicted pairwise treatment effect. Two patient pairs are uninformative if the pairs have the same observed pairwise treatment effect. The C-for-benefit is 0.5 if the model cannot distinguish between patients any better than random treatment assignment, and 1 if the model can perfectly distinguish between patients who benefit from treatment and who do not.

#### Overall performance measures

We propose to measure overall performance, i.e., the accuracy of individualized treatment effect estimates, using the multi-class versions of the Brier score and cross-entropy because observed pairwise treatment effect can belong to one of three classes (benefit, no effect, harm) [11, 12]. We defined cross-entropy-for-benefit as the logarithmic distance between predicted and observed pairwise treatment effect and Brier-for-benefit as the average squared distance between predicted and observed pairwise treatment effect (Additional file 2). Thus, the overall performance metrics indicate better optimal performance when closer to zero. The cross-entropy-for-benefit and Brier-for-benefit measure overall model performance since these metrics are affected by calibration and discrimination simultaneously.

### Data

To illustrate the proposed metrics, we used data from the Diabetes Prevention Program (DPP). The participants of DPP were at risk to develop diabetes, which is defined as a body mass index of 24 or higher and impaired glucose metabolism [19]. The participants were randomized between 1996 and 2001 to receive 1) an intensive program of lifestyle modification lessons, 2) 850 mg of metformin twice a day and standard lifestyle modification, or 3) placebo twice a day and standard lifestyle recommendations. To predict the effect of the intervention on the outcome, i.e., the risk of developing diabetes, we used the patient characteristics sex, age, ethnicity, body mass index, smoking status, fasting blood sugar, triglycerides, hemoglobin, self-reported history of hypertension, family history of diabetes, self-reported history of high blood glucose, and gestational diabetes mellitus (Additional file 3). We imputed missing values of patient characteristics using Multivariate Imputations by Chained Equations [20].

### Simulation study

We simulated the outcomes of the DPP using the patient characteristics to study if the proposed performance metrics were better for the model used for outcome generation (“optimal model”) than for deliberately “perturbed models”. The “optimal model” was a logistic regression model for the probability of the outcome (developing diabetes) ${p}_{i}$ based on the treatment (e.g., lifestyle intervention) assignment indicator $W$, standardized patient characteristics $X$, and their interaction:

$$\mathrm{log}\frac{{p}_{i}}{1-{p}_{i}}={W}_{i}\cdot {\beta }_{W}+X\cdot {\beta }_{X}+{W}_{i}\cdot X\cdot {\beta }_{W\cdot X}.$$

The regression coefficients of this model were obtained through Ridge regression on the original data set (see Additional file 4 for the penalty factor).

Next, we created a super population by duplicating the matched patient pairs 300 times to obtain high precision to ensure that observed differences between metrics are “true” differences. The outcomes of the super population ${Y}_{i}$ were simulated from a Bernoulli distribution with the outcome probabilities ${p}_{i}$ generated by the “optimal model”.

We then created three deliberate perturbations of the “optimal model”. The first “perturbed model” overestimates ATE by multiplying the coefficient of the treatment assignment indicator $\left({\beta }_{W}\right)$ with 2 (Additional files 5 and 6). Additionally, we perturbed a model that underestimates ATE by multiplying the coefficient of the treatment assignment indicator $\left({\beta }_{W}\right)$ with 0.5. The second “perturbed model” overestimates risk heterogeneity by multiplying the coefficient of the patient characteristics $\left({\beta }_{X}\right)$ by 2 (Additional files 5 and 6). The third “perturbed model” overestimates treatment effect heterogeneity by multiplying the coefficient of the interaction between treatment assignment and the patient characteristics $\left({\beta }_{W\cdot X}\right)$ by 3 (Additional files 5 and 6). We calculated the root mean squared error (RMSE) to indicate the level of perturbation for each model.

Finally, we computed the performance metrics for the “optimal” and the three “perturbed models” in the super population. We also visualized the performance of each of the four models with treatment effect calibration plots.

### Case study

The performance of three different modelling approaches to predict treatment effect for patients at risk of diabetes in the DPP data set was compared using the proposed metrics.

The first approach (“risk model”) uses logistic regression to explain the outcome probability ${p}_{i}=P\left({Y}_{i}=1|{X}_{i}=x,{W}_{i}=w\right)$ based on the treatment indicator $W$, the centered prognostic index $PI$, and their interaction:

$$\mathrm{log}\frac{{p}_{i}}{1-{p}_{i}}=W\cdot {\beta }_{W}+s(PI)\cdot {\beta }_{PI}+W\cdot s(PI)\cdot {\beta }_{W\cdot PI},$$

where $s(\cdot )$ represents restricted cubic splines with two degrees of freedom, and $PI$ was determined by regressing the outcome variable on the patient characteristics $X (PI={X}^{^{\prime}}{\widehat{\beta }}_{X})$.

The second approach (“effect model”) uses a penalized Ridge logistic regression to explain the outcome probability ${p}_{i}$ based on the unpenalized treatment indicator $W$, penalized centered patient characteristics $X$, and their penalized interaction:

$$\mathrm{log}\frac{{p}_{i}}{1-{p}_{i}}=W\cdot {\beta }_{W}+X\cdot {\beta }_{X}+W\cdot X\cdot {\beta }_{W\cdot X},$$

where the level of penalization was determined by the minimum squared error in 5-fold cross-validation [21] (Additional file 4).

The third approach is a causal forest, which is similar to a random forest but maximizes heterogeneity in treatment effect rather than variation in the outcome [22]. Causal trees were built honestly by partitioning the data into two subsamples. One subsample was used to construct the trees, and another subsample to predict the treatment effect [22]. The parameters of the causal forest were tuned, of which we made the specifics available in Additional file 4.

To mimic external validation, the models were trained on 70 percent of the patient data. The remaining 30 percent of the patient data, the test set, was used to calculate performance metrics with confidence intervals using 100 bootstrap samples of matched patient pairs [23]. We used the R packages MatchIt for matching patients, mice for single imputation, stats for local regression, rms for restricted cubic splines, glmnet for Ridge penalization, and grf for causal forest (R version 4.1.0) [20, 24–28].

## Results

### Patient data

Between 1996 and 2001, the DPP collected data on 3,081 participants of which 1,024 received lifestyle intervention, 1,027 received metformin, and 1,030 received placebo treatment (Additional file 3). The median age of the participants was 52 years (IQR: 42–57 years), 67% of the participants were female, and the median BMI value was 33 (IQR: 29–37). The proportion of patients developing diabetes was 4.8%, 7.0%, and 9.5% among participants receiving lifestyle intervention, metformin, and placebo treatment, respectively (Additional file 3).

### Simulation study

**Fig. 2:**
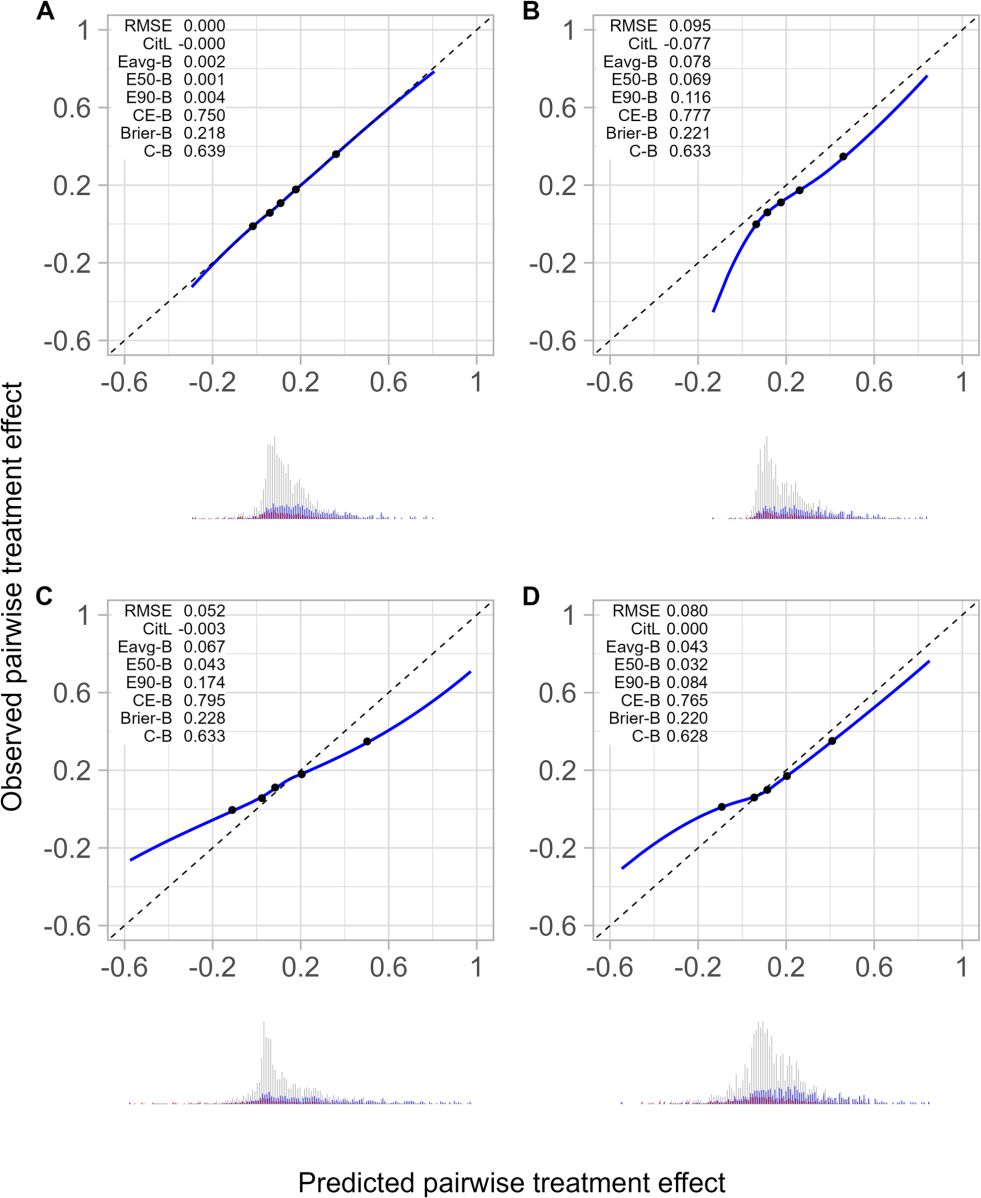
Calibration plots of pairwise treatment effect of simulated data from patients receiving lifestyle intervention. This Figure depicts observed versus predicted pairwise treatment effect by smoothed calibration curves (blue line) and quantiles of predicted pairwise treatment effect (black dots) of simulated data from the lifestyle intervention versus placebo treatment. Observed pairwise treatment effect was obtained by matching patients based on patient characteristics. Smoothed calibration curves were obtained by local regression of the observed pairwise treatment effect of matched patient pairs on predicted pairwise treatment effect of matched patient pairs. For prediction of individualized treatment effect, we used a treatment effect modelling approach for the “optimal model” (panel **A**) and three “perturbed models” that overestimate average treatment effect (panel **B**), risk heterogeneity (panel **C**), and treatment effect heterogeneity (panel **D**). The average treatment effect is 13.0, 20.9, 13.0 (after a correction of ${\beta }_{W}$ with -0.195), and 13.0 (after a correction of ${\beta }_{W}$ with -0.19), respectively. Abbreviations: RMSE, root mean squared error; CitL, calibration-in-the-large; Eavg-B, E_avg_-for-benefit; E50-B, E_50_-for-benefit; E90-B, E_90_-for-benefit; CE-B, cross-entropy-for-benefit; Brier-B, Brier-for-benefit; C-B, C-for-benefit

As expected, the treatment effect predictions of the “optimal model” were almost perfectly calibrated (calibration-in-the-large = 0.000, E_avg_-for-benefit = 0.002, E_50_-for-benefit = 0.001, E_90_-for-benefit = 0.004, Fig. 2A). The “optimal model” was well able to discriminate (C-for-benefit = 0.639, Fig. 2A) between patients with small treatment harm (ATE = -0.017 in the quantile of patients with smallest predicted pairwise treatment effect) and patients with substantial treatment benefit (ATE = 0.361 in the quantile of patients with largest treatment effect).

The first “perturbed model” was designed to overestimate treatment effect of lifestyle intervention (RMSE = 0.095), which was expressed graphically by the corresponding calibration curve lying below the 45-degree line, and numerically by suboptimal calibration metrics (calibration-in-the-large = -0.077, E_avg_-for-benefit = 0.078, E_50_-for-benefit = 0.069, E_90_-for-benefit = 0.116, Fig. 2B). The C-for-benefit expressed a slightly poorer ability to distinguish between patients with small and large treatment effects than the “optimal model” (C-for-benefit = 0.633 versus 0.639). The cross-entropy-for-benefit and Brier-for-benefit also expressed poorer overall performance than the “optimal model” (cross-entropy-for-benefit = 0.777 versus 0.750, Brier-for-benefit = 0.221 versus 0.218, Fig. 2A and B). When underestimating treatment effect of lifestyle intervention, the proposed metrics correctly identified that the performance of the data-generating model was better than the perturbed model (Additional file 7).

The second “perturbed model” was designed to overestimate risk heterogeneity of patients receiving lifestyle intervention (RMSE = 0.052), which was expressed graphically by the corresponding calibration curve lying above the diagonal for low predicted pairwise treatment effect (underestimation of low treatment effect) and below the diagonal for high predicted pairwise treatment effect (overestimation of high treatment effect), and numerically by suboptimal calibration metrics (calibration-in-the-large = -0.003, E_avg_-for-benefit = 0.067, E_50_-for-benefit = 0.043, E_90_-for-benefit = 0.174, Fig. 2C). The C-for-benefit expressed a slightly poorer ability to distinguish between patients with small and large treatment effects than the “optimal model” (C-for-benefit = 0.633 versus 0.639). The cross-entropy-for-benefit and Brier-for-benefit also expressed poorer overall performance than the “optimal model” (cross-entropy-for-benefit = 0.795 versus 0.750, Brier-for-benefit = 0.228 versus 0.218, Fig. 2A and C).

The third “perturbed model” was designed to overestimate treatment effect heterogeneity of patients receiving lifestyle intervention (RMSE = 0.080), which was expressed graphically by the corresponding calibration curve lying more extremely above the diagonal for low predicted pairwise treatment effect (underestimation of low treatment effect) and more extremely below the diagonal for high predicted pairwise treatment effect (overestimation of high treatment effect), and numerically by suboptimal calibration metrics (E_avg_-for-benefit = 0.043, E_50_-for-benefit = 0.032, E_90_-for-benefit = 0.084, Fig. 2D). The C-for-benefit expressed a slightly poorer ability to distinguish between patients with small and large treatment effects than the “optimal model” (C-for-benefit = 0.628 versus 0.639, Fig. 2D). The cross-entropy-for-benefit and Brier-for-benefit also expressed poorer overall performance than the “optimal model” (cross-entropy-for-benefit = 0.765 versus 0.750, Brier-for-benefit = 0.220 versus 0.218, Fig. 2A and D).

The results from the simulations using the metformin treatment arm rather than the lifestyle intervention arm were similar (Fig. 2; Additional file 8).

### Case study

**Fig. 3:**
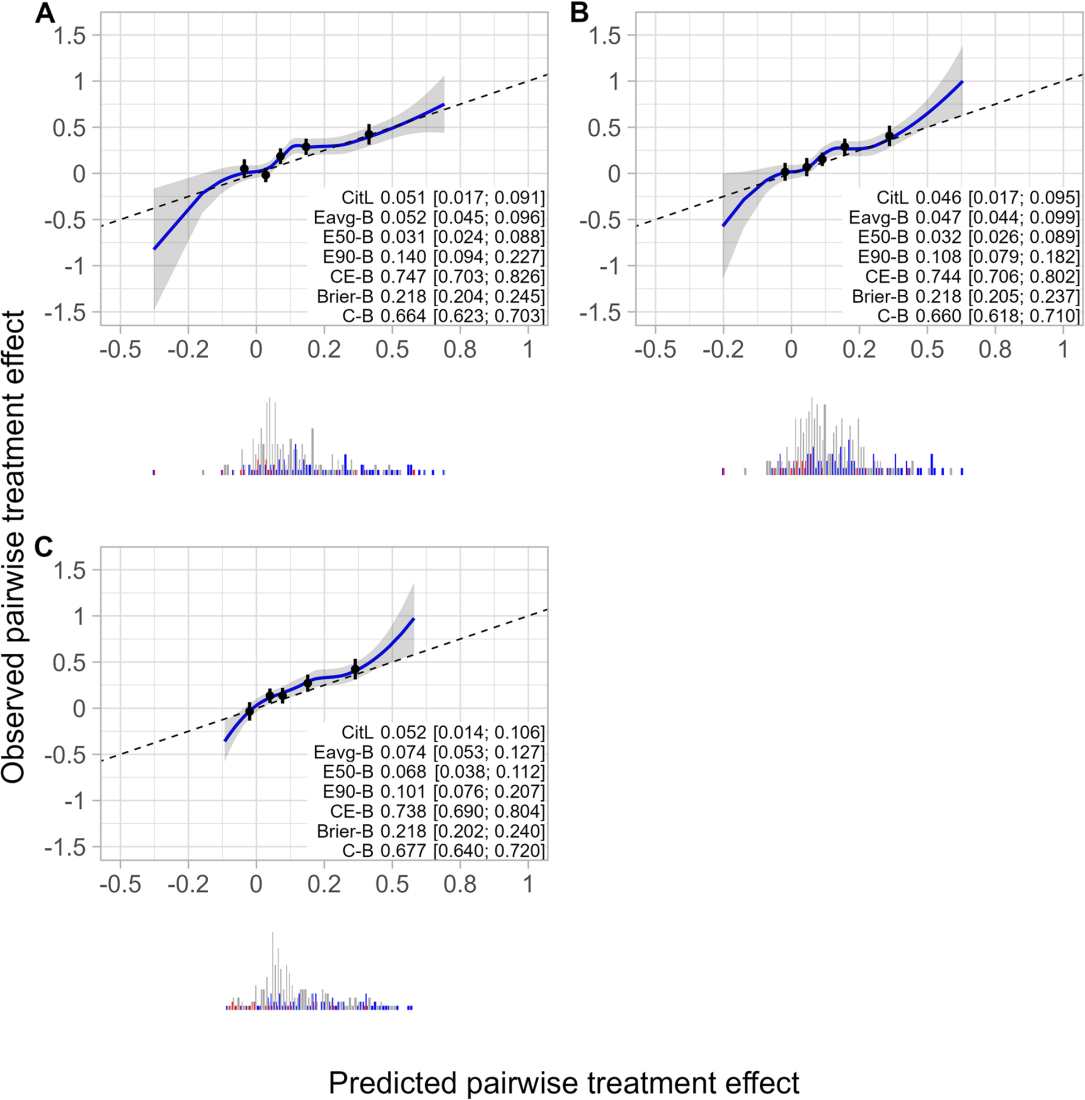
Calibration plot of pairwise treatment effect of DPP data from patients receiving lifestyle intervention. This Figure depicts observed versus predicted pairwise treatment effect by smoothed calibration curves (blue line with 95% confidence interval displayed by grey shaded area) and quantiles of predicted pairwise treatment effect (black dots) of lifestyle intervention versus placebo treatment. Observed pairwise treatment effect was obtained by matching patients based on patient characteristics. Smoothed calibration curves were obtained by local regression of the observed pairwise treatment effect of matched patient pairs on predicted pairwise treatment effect of matched patient pairs. For prediction of individualized treatment effect, we used: a risk modelling approach (panel **A**), a treatment effect modelling approach (panel **B**), and a causal forest (panel **C**). Confidence intervals around metric values were obtained using 100 bootstrap samples. Abbreviations: CitL, calibration-in-the-large; Eavg-B, E_avg_-for-benefit; E50-B, E_50_-for-benefit; E90-B, E_90_-for-benefit; CE-B, cross-entropy-for-benefit; Brier-B, Brier-for-benefit; C-B, C-for-benefit

The differences in any of the performance measures between the risk model, the effect model, and the causal forest were not significantly different from zero in the 30 percent of patients who were in the test dataset (n = 617; Additional file 3). Numerically, most calibration metrics of the effect model were better than that of the risk model (calibration-in-the-large = 0.046 versus 0.051; E_avg_-for-benefit = 0.047 versus 0.052; E_90_-for-benefit = 0.108 versus 0.140, Fig. 3A and B). Consequently, the overall performance of the effect model was numerically better than that of the risk model (cross-entropy-for-benefit = 0.744 versus 0.747, Fig. 3A and B), despite the numerically poorer discriminative ability of the effect model (C-for-benefit = 0.660 versus 0.664, Fig. 3A and B).

Central calibration metrics of the causal forest were numerically poorer than those of the risk model (calibration-in-the-large = 0.052 versus 0.051; E_avg_-for-benefit = 0.074 versus 0.052; E_50_-for-benefit = 0.068 versus 0.031, Fig. 3A and C), but the causal forest resulted in less extreme miscalibration than the risk model (E_90_-for-benefit = 0.101 versus 0.140, Fig. 3A and C). Due to less extreme miscalibration and numerically better discriminative ability (C-for-benefit = 0.677 versus 0.664, Fig. 3A and C), the overall performance of the causal forest was numerically better than that of the risk model (cross-entropy-for-benefit = 0.738 versus 0.747, Fig. 3A and C). In the sensitivity analysis, the values and model preferences of the proposed metrics slightly changed when using different matching procedures (Additional file 1).

## Discussion

We extended the E-statistics, cross-entropy, and Brier score to quantify the quality of treatment effect predictions in RCTs. As shown in the illustration in Fig. 1, the proposed metrics assessed performance of models predicting individualized treatment effect more effectively than conventional metrics developed to assess performance of models predicting risk. The simulation study showed that the proposed metrics correctly identified that the performance of the data-generating model was consistently better than those of deliberately “perturbed models”. The case study illustrated the use of the proposed metrics in practice and showed that the calibration, discriminative ability, and overall performance of the three different models predicting treatment effect were not significantly different.

Similar to the previously proposed C-for-benefit, we defined observed pairwise treatment effect by the difference between outcomes in pairs of matched patients [9]. Matching patients based on predicted pairwise treatment effect would result in different patient pairs and consequently different observed pairwise treatment effect for each prediction model [9]. Therefore, we chose to match patients based on the Mahalanobis distance between patient characteristics resulting in the same observed pairwise treatment effect for each prediction model to allow for model comparison. The predicted pairwise treatment effect when matching patients by patient characteristics is more heterogeneous than the individual predicted treatment effect resulting from the model, which is more apparent in a smaller sample. We matched without replacement since the treatment arms were similar in size, but matching with replacement is more appropriate for samples with unbalanced treatment arms. Future research is needed to investigate the use of the proposed metrics for models predicting individualized treatment effect in observational data. The matching of patients may correct for (measured) confounders when estimating an average treatment effect in observational data, but simulations with confounders are required to understand if the performance metrics are useful for the comparison of treatment effect prediction models. Furthermore, we selected relevant patient characteristics based on clinical expertise and existing literature, but variable selection is more suitable in high-dimensionality data. The proposed metrics varied slightly when choosing different matching procedures. However, the purpose of this study is not to determine an optimal matching strategy, but to propose metrics for evaluation of models predicting individualized treatment effect. Further research should more extensively investigate the influence of different matching procedures on the proposed metrics.

The case study is merely an illustration of the use of the performance metrics and not a framework for model selection or internal validation. The use of internal validation techniques other than split sampling is recommended for quantification of the performance of a model in similar settings, but that was outside the scope of this study [29]. The choice of the percentage of observations used for the training and test set was arbitrary. Furthermore, the proposed metrics in the training set will not be insightful when using models with penalization and honest tree building, because they will indicate by definition miscalibration in the training set (Additional files 9 and 10). Additionally, we did not calculate the proposed metrics in the training set (panel A; C; E in Additional files 9 and 10), because these would be apparent values and need to be corrected for optimism since the model was developed in the same data.

The strength of our study is that we propose currently lacking performance metrics for models predicting treatment effect. Their actual values can be used to compare models predicting treatment effect. Furthermore, in future research updating strategies can be considered if our proposed calibration metrics indicate miscalibration of treatment effect predictions.

A limitation of this study is the limited sample size of the case study. In the simulation study, we showed that the performance metrics were able to distinguish between models for an artificially enlarged data set. However, in the case study, the confidence intervals of the performance metrics were overlapping. This phenomenon is inherent to treatment effect estimation. To obtain reasonable power, treatment effect analyses require a much larger sample size compared to when estimating an overall ATE [30]. The case study suggested that there is a trade-off between calibration and discrimination: better calibrated models were worse at discriminating between patients with small and large treatment effects, but due to the small sample size no strict conclusions can be drawn. Secondly, the performance metrics were developed for binary outcomes, which could be extended to continuous outcomes in future research. Notwithstanding these limitations, we conclude that the proposed metrics are useful to assess the calibration and overall performance of models predicting treatment effect in RCTs.

## Conclusions

We showed that the proposed metrics are useful to assess and compare the calibration and overall performance of models designed to predict treatment effect in RCTs.

## Supplementary Information

**Additional file 1.** Sensitivity analysis of matching method.

**Additional file 2.** Derivation of the metrics measuring overall performance of models predicting treatment effect.

**Additional file 3.** Characteristics of patients in the Diabetes Prevention Program receiving lifestyle intervention, metformin, or placebo treatment.

**Additional file 4.** Parameter settings of Ridge regression and causal forest.

**Additional file 5.** The probability of the potential outcome of diabetes under lifestyle intervention versus under control treatment predicted by the “optimal model”, and three “perturbed models” that overestimate average treatment effect, risk heterogeneity, and treatment effect heterogeneity.

**Additional file 6.** The probability of the potential outcome of diabetes under metformin treatment versus under control treatment predicted by the “optimal model”, and three “perturbed models” that overestimate average treatment effect, risk heterogeneity, and treatment effect heterogeneity.

**Additional file 7.** Calibration plots of pairwise treatment effect of simulated data from patients receiving lifestyle intervention or metformin. This Figure depicts observed versus predicted pairwise treatment effect by smoothed calibration curves (blue line) and quantiles of predicted pairwise treatment effect (black dots) of simulated data from the lifestyle intervention or metformin versus placebo treatment. Observed pairwise treatment effect was obtained by matching patients based on patient characteristics. Smoothed calibration curves were obtained by local regression of the observed pairwise treatment effect of matched patient pairs on predicted pairwise treatment effect of matched patient pairs. For prediction of individualized treatment effect, we used the “perturbed model” that underestimates average treatment effect for lifestyle intervention (panel A) and metformin treatment (panel B). The average treatment effect is 7.1 and 3.5, respectively.

**Additional file 8.** Calibration plots of pairwise treatment effect of simulated data from patients receiving metformin intervention. This Figure depicts observed versus predicted pairwise treatment effect by smoothed calibration curves (blue line) and quantiles of predicted pairwise treatment effect (black dots) of simulated data from the metformin versus placebo treatment. Observed pairwise treatment effect was obtained by matching patients based on patient characteristics. Smoothed calibration curves were obtained by local regression of the observed pairwise treatment effect of matched patient pairs on predicted pairwise treatment effect of matched patient pairs. For prediction of individualized treatment effect, we used a treatment effect modelling approach for the “optimal model” (panel A) and three “perturbed models” that overestimate average treatment effect (panel B), risk heterogeneity (panel C), and treatment effect heterogeneity (panel D). The average treatment effect is 6.6, 11.9, 6.6 (after a correction of with -0.085), and 6.6 (after a correction of with -0.16), respectively.

**Additional file 9.** Calibration plot of pairwise treatment effect of training and test data of lifestyle intervention. This Figure depicts observed versus predicted pairwise treatment effect by smoothed calibration curves (blue line with 95% confidence interval displayed by grey shaded area) and quarters of predicted pairwise treatment effect (black dots) of lifestyle intervention versus placebo treatment. Observed pairwise treatment effect was obtained by matching patients based on patient characteristics. Smoothed calibration curves were obtained by local regression of the observed pairwise treatment effect of matched patient pairs on predicted pairwise treatment effect of matched patient pairs. For prediction of treatment effect, we used: a risk modelling approach (panel A; B), a treatment effect modelling approach (panel C; D), and a causal forest (panel E; F). The models are trained on 70 percent of the data (panel A; C; E) and evaluated on the other 30 percent of the data (B; D; F). Confidence intervals around the metric values were obtained using 100 bootstrap samples.

**Additional file 10.** Calibration plot of pairwise treatment effect of training and test data of metformin intervention. This Figure depicts observed versus predicted pairwise treatment effect by smoothed calibration curves (blue line with 95% confidence interval displayed by grey shaded area) and quarters of predicted pairwise treatment effect (black dots) of metformin versus placebo treatment. Observed pairwise treatment effect was obtained by matching patients based on patient characteristics. Smoothed calibration curves were obtained by local regression of the observed pairwise treatment effect of matched patient pairs on predicted pairwise treatment effect of matched patient pairs. For prediction of treatment effect, we used: a risk modelling approach (panel A; B), a treatment effect modelling approach (panel C; D), and a causal forest (panel E; F). The models are trained on 70 percent of the data (panel A; C; E) and evaluated on the other 30 percent of the data (B; D; F). Confidence intervals around the metric values were obtained using 100 bootstrap samples.

## Data Availability

The dataset supporting the conclusions of this article is available in the NIDDK Repository website (https://repository.niddk.nih.gov/studies/dpp/). Project home page: https://github.com/CHMMaas/PaperPredictionMetrics.

## Abbreviations

ATE: Average treatment effect
DPP: Diabetes Prevention Program
IQR: Interquartile Range
RMSE: Root mean squared error
