# Supplementary material for: Performance metrics for models designed to predict treatment effect
Source: BMC Med Res Methodol. 2023 Jul 8;23:165. doi: 10.1186/s12874-023-01974-w (PMC10329397; doi:10.1186/s12874-023-01974-w)
Supplement: Supplementary file 1 — Additional file 1. Sensitivity analysis of matching method. [file 12874_2023_1974_MOESM1_ESM.docx]

**Additional file 1**.

We match patients that are nearest neighbours based on the Mahalanobis distance between patient characteristics without replacement. We consider two other matching procedures, varying (i) the distance to robust Mahalanobis distance or (2) method to optimal matching. Robust Mahalanobis distance is computed not on the covariates directly, but on their ranks and it uses a correction for ties[1]. Optimal pair matching is similar to nearest neighbour matching, but instead of greedily matching the nearest neighbour it matches such that the sum of the absolute pairwise distances in the matched sample is as small as possible[2]. As a result, a matching order is not required and it is less likely that extreme within-pair distances will be large, unlike with nearest neighbour matching.

**Performance of models predicting the probability of developing diabetes when treated with lifestyle treatment using three different matching procedures.** Abbreviation: ATE, average treatment effect.

| *Method* | *Nearest* | | | *Nearest* | | | *Optimal* | | |
| --- | --- | --- | --- | --- | --- | --- | --- | --- | --- |
| *Distance* | *Mahalanobis* | | | *Robust Mahalanobis* | | | *Mahalanobis* | | |
| *Replacement* | *No replacement* | | | *No replacement* | | | *No replacement* | | |
| ***Measures*** | Risk | Effect | CF | Risk | Effect | CF | Risk | Effect | CF |
| *ATE* | 0.212 | 0.217 | 0.221 | 0.212 | 0.217 | 0.221 | 0.212 | 0.217 | 0.221 |
| *Pairwise ATE* | 0.135 | 0.140 | 0.135 | 0.124 | 0.128 | 0.129 | 0.132 | 0.137 | 0.133 |
| *Calibration-in-the-large* | 0.051 | 0.047 | 0.051 | 0.066 | 0.061 | 0.061 | 0.058 | 0.053 | 0.057 |
| *Eavg-for-benefit* | 0.052 | 0.046 | 0.066 | 0.079 | 0.068 | 0.078 | 0.076 | 0.077 | 0.063 |
| *E50-for-benefit* | 0.031 | 0.040 | 0.064 | 0.077 | 0.075 | 0.075 | 0.063 | 0.068 | 0.073 |
| *E90-for-benefit* | 0.140 | 0.080 | 0.107 | 0.117 | 0.098 | 0.125 | 0.145 | 0.128 | 0.096 |
| *Cross-entropy-for-benefit* | 0.747 | 0.741 | 0.743 | 0.737 | 0.730 | 0.739 | 0.709 | 0.707 | 0.717 |
| *Brier-for-benefit* | 0.218 | 0.217 | 0.219 | 0.214 | 0.214 | 0.215 | 0.202 | 0.203 | 0.208 |
| *C-for-benefit* | 0.664 | 0.664 | 0.674 | 0.620 | 0.631 | 0.644 | 0.606 | 0.609 | 0.631 |

**Performance of models predicting the probability of developing diabetes when treated with metformin treatment using three different matching procedures.** Abbreviation: ATE, average treatment effect.

| *Method* | *Nearest* | | | *Nearest* | | | *Optimal* | | |
| --- | --- | --- | --- | --- | --- | --- | --- | --- | --- |
| *Distance* | *Mahalanobis* | | | *Robust Mahalanobis* | | | *Mahalanobis* | | |
| *Replacement* | *No replacement* | | | *No replacement* | | | *No replacement* | | |
| ***Measures*** | Risk | Effect | CF | Risk | Effect | CF | Risk | Effect | CF |
| *ATE* | 0.244 | 0.243 | 0.269 | 0.244 | 0.243 | 0.269 | 0.244 | 0.243 | 0.269 |
| *Pairwise ATE* | 0.084 | 0.085 | 0.078 | 0.080 | 0.083 | 0.075 | 0.082 | 0.083 | 0.075 |
| *Calibration-in-the-large* | 0.034 | 0.033 | 0.040 | 0.041 | 0.038 | 0.045 | 0.036 | 0.035 | 0.042 |
| *Eavg-for-benefit* | 0.051 | 0.046 | 0.067 | 0.051 | 0.044 | 0.049 | 0.044 | 0.068 | 0.049 |
| *E50-for-benefit* | 0.052 | 0.026 | 0.050 | 0.057 | 0.023 | 0.028 | 0.015 | 0.049 | 0.017 |
| *E90-for-benefit* | 0.072 | 0.105 | 0.135 | 0.074 | 0.100 | 0.153 | 0.135 | 0.188 | 0.178 |
| *Cross-entropy-for-benefit* | 0.766 | 0.767 | 0.786 | 0.762 | 0.761 | 0.775 | 0.773 | 0.776 | 0.784 |
| *Brier-for-benefit* | 0.222 | 0.222 | 0.228 | 0.220 | 0.220 | 0.222 | 0.224 | 0.225 | 0.227 |
| *C-for-benefit* | 0.660 | 0.679 | 0.695 | 0.657 | 0.669 | 0.664 | 0.657 | 0.677 | 0.673 |

The **original matching procedure** uses nearest-neighbour matching using Mahalanobis distance without replacement. For prediction of individualized treatment effect of lifestyle intervention, we used: a risk modelling approach (panel A), a treatment effect modelling approach (panel B), and a causal forest (panel C).


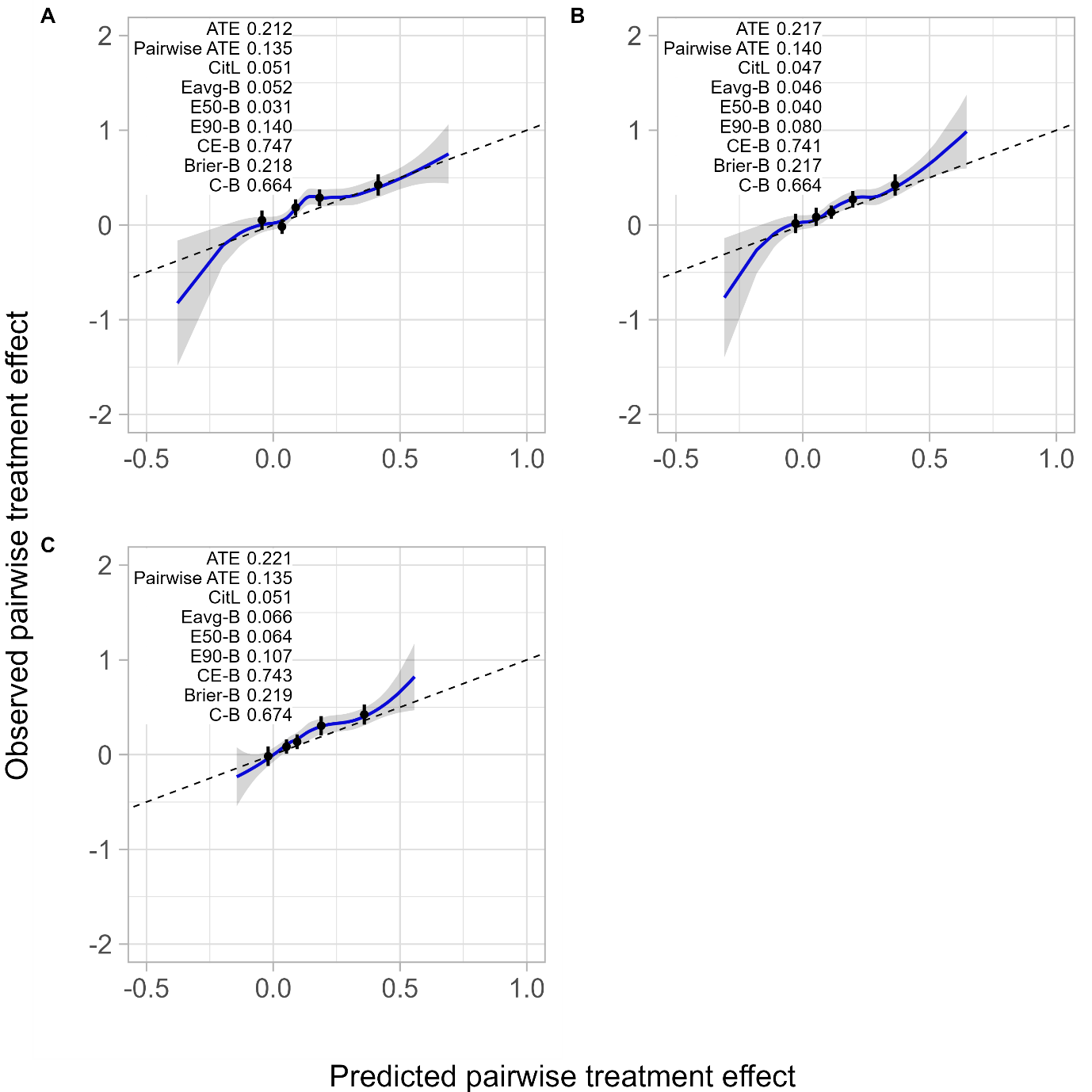


**Alternative matching scenario 1** uses nearest-neighbour matching using robust Mahalanobis distance without replacement. For prediction of individualized treatment effect of lifestyle intervention, we used: a risk modelling approach (panel A), a treatment effect modelling approach (panel B), and a causal forest (panel C).


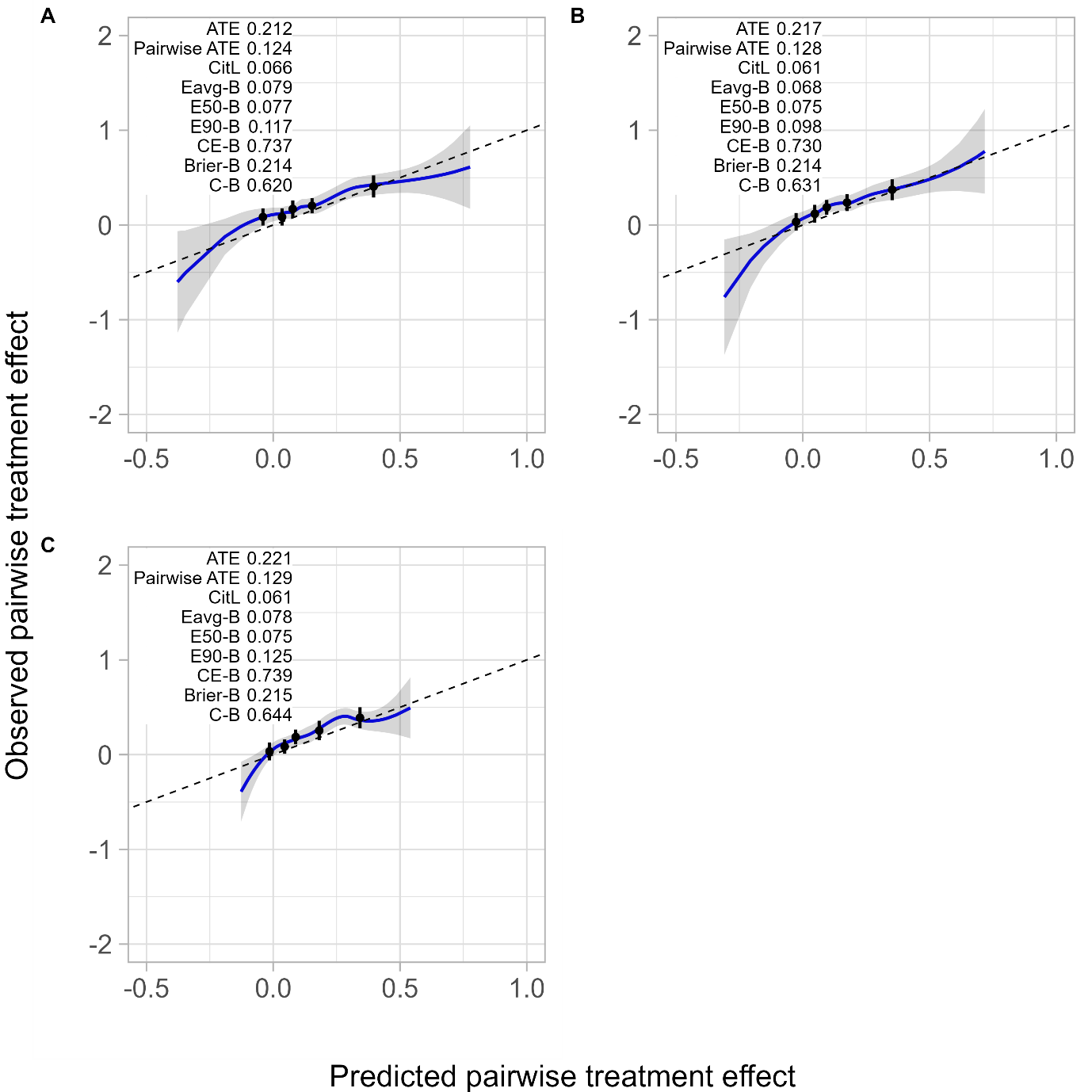


**Alternative matching scenario 2** uses optimal matching using Mahalanobis distance without replacement. For prediction of individualized treatment effect of lifestyle intervention, we used: a risk modelling approach (panel A), a treatment effect modelling approach (panel B), and a causal forest (panel C).


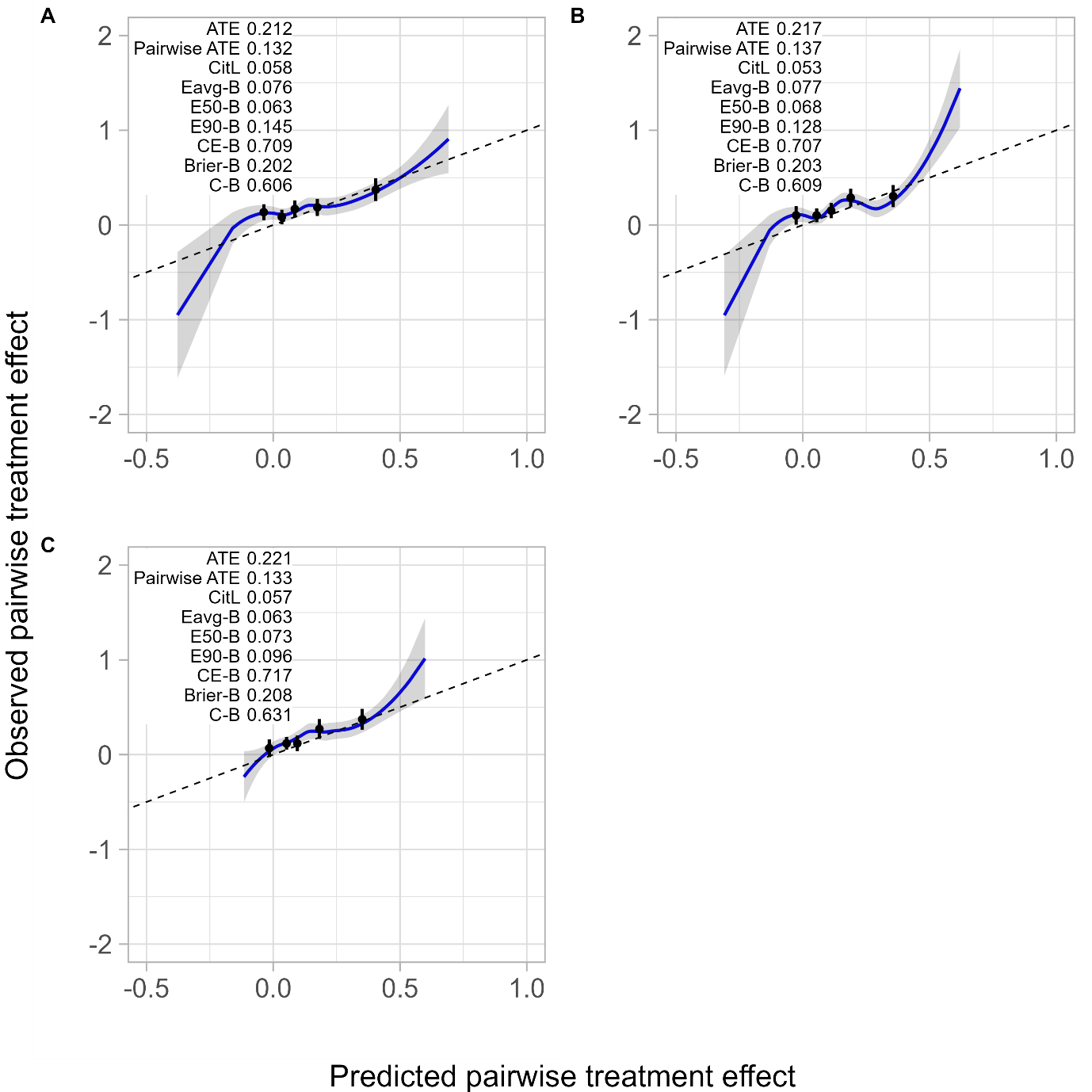


**References**

1. Rosenbaum PR: **Design of Observational Studies**: Springer; 2010.

2. Hansen BB, Olsen Klopfer S: **Optimal Full Matching and Related Designs via Network Flows**. *Journal of Computational and Graphical Statistics* 2006, **15**(3):609-627.

3. Abadie A, Imbens GW: **On the Failure of Bootstrap for Matching Estimators**. *Econometrica* 2008, **76**(6):1537-1557.
