# Supplementary material for: Performance metrics for models designed to predict treatment effect
Source: BMC Med Res Methodol. 2023 Jul 8;23:165. doi: 10.1186/s12874-023-01974-w (PMC10329397; doi:10.1186/s12874-023-01974-w)
Supplement: Supplementary file 2 — Additional file 2. Derivation of the metrics measuring overall performance of models predicting treatment effect. [file 12874_2023_1974_MOESM2_ESM.docx]

**Additional file 2. Derivation of the metrics measuring overall performance of models predicting treatment effect.**

*Derivation of the Brier-for-benefit*

The Brier-for-benefit is defined as

Brier-for-benefit$=\frac{1}{2n_{p}}\sum_{i=1}^{n_{p}} \sum_{c\in\left\{ -1,0,1 \right\}} \left( P\left( \tau_{i}=c \right)-I\left( \tau_{i}=c \right) \right)^{2},$

where $n_{p}$ indicates the number of pairs, $\tau_{i}$ indicates the observed pairwise treatment effect in a matched pair $i$, $I\left( \tau_{i}=c \right)$ is an indicator function returning one when the observed pairwise treatment effect of matched pair $i$ $\left( \tau_{i} \right)$ is equal to class $c$, and $P(\tau_{i}=c)$ indicates the probability that the observed pairwise treatment effect of matched pair $i$ is equal to class $c$. The Brier score is divided by two to ensure that it lies between zero and one because in the worst-case scenario you give the highest prediction (one) for the wrong class, which would give a Brier score of two. Equivalently,

Brier-for-benefit$=\frac{1}{2n_{p}}\sum_{i=1}^{n_{p}} \left[ \left( P\left( \tau_{i}=1 \right)-I\left( \tau_{i}=1 \right) \right)^{2}+\left( P\left( \tau_{i}=0 \right)-I\left( \tau_{i}=0 \right) \right)^{2}+\left( P\left( \tau_{i}=-1 \right)-I\left( \tau_{i}=-1 \right) \right)^{2} \right]$

Since matched patient pairs are independent, it holds that

$$P\left( \tau_{i}=1 \right)=P\left( Y_{i}\left( 1 \right)=0, Y_{i}\left( 0 \right)=1 \right)$$

$$=P\left( Y_{i}\left( 1 \right)=0 \right)P\left( Y_{i}\left( 0 \right)=1 \right)$$

$$=\left( 1-p_{i,1} \right)p_{i,0}$$

$$P\left( \tau_{i}=0 \right)=P\left( \left( Y_{i}\left( 1 \right)=0, Y_{i}\left( 0 \right)=0 \right)\cap\left( Y_{i}\left( 1 \right)=1, Y_{i}\left( 0 \right)=1 \right) \right)$$

$$=P\left( Y_{i}\left( 1 \right)=0 \right)P\left( Y_{i}\left( 0 \right)=0 \right)+P\left( Y_{i}\left( 1 \right)=1 \right)P\left( Y_{i}\left( 0 \right)=1 \right)$$

$$=\left( 1-p_{i,1} \right)\left( 1-p_{i,0} \right)+p_{i,1}p_{i, 0}$$

$$P\left( \tau_{i}=-1 \right)=P\left( Y_{i}\left( 1 \right)=1, Y_{i}\left( 0 \right)=0 \right)$$

$$=P\left( Y_{i}\left( 1 \right)=1 \right)P\left( Y_{i}\left( 0 \right)=0 \right)$$

$$=p_{i,1}\left( 1-p_{i,0} \right),$$

where $Y_{i}\left( W_{i} \right)=\left\{ \begin{aligned} Y_{i}\left( 0 \right) \mathrm{if} W_{i}=0 \\ Y_{i}\left( 1 \right) \mathrm{if} W_{i}=1 \end{aligned} \right.$ with $Y_{i}$ indicates the potential outcome for patient $i$ and $W_{i}$ indicates the binary indicator for treatment, and the outcome probabilities conditional on treatment

$$p_{i,1}=P\left( Y_{i}\left( 1 \right)=1 \right)=P\left( Y_{i}=1 | W_{i}=1 \right)$$

$$p_{i,0}=P\left( Y_{i}\left( 0 \right)=1 \right)=P\left( Y_{i}=1 | W_{i}=0 \right).$$

As a result, the Brier-for-benefit can be expressed as

Brier-for-benefit$=\frac{1}{2n_{p}}\sum_{i=1}^{n_{p}} \left( \left( 1-p_{i,1} \right)p_{i,0}-I\left( \tau_{i}=1 \right) \right)^{2}$

$$=+\frac{1}{2n_{p}}\sum_{i=1}^{n_{p}} \left( \left( 1-p_{i,1} \right)\left( 1-p_{i,0} \right)+p_{i,1}p_{i,0}-I\left( \tau_{i}=0 \right) \right)^{2}$$

$$=+\frac{1}{2n_{p}}\sum_{i=1}^{n_{p}} \left( p_{i,1}\left( 1-p_{i,0} \right)-I\left( \tau_{i}=-1 \right) \right)^{2}.$$

*Derivation of the cross-entropy-for-benefit*

Similarly, the cross-entropy-for-benefit is defined as

Cross-entropy-for-benefit $=-\frac{1}{n_{p}}\cdot\sum_{i=1}^{n_{p}} \sum_{c\in\left\{ -1,0,1 \right\}} I\left( \tau_{i}=c \right)\log\left[ P\left( \tau_{i}=c \right) \right]$

$$=-\frac{1}{n_{p}}\cdot\sum_{i=1}^{n_{p}} I\left( \tau_{i}=1 \right)\log\left[ \left( 1-p_{i,1} \right)p_{i,0} \right]$$

$$-\frac{1}{n_{p}}\cdot\sum_{i=1}^{n_{p}} I\left( \tau_{i}=0 \right)\log\left[ \left( 1-p_{i,1} \right)\left( 1-p_{i,0} \right)+p_{i,1}p_{i,0} \right]$$

$$-\frac{1}{n_{p}}\cdot\sum_{i=1}^{n_{p}} I\left( \tau_{i}=-1 \right)\log\left[ p_{i,1}\left( 1-p_{i,0} \right) \right].$$

*Outcome probabilities of the causal forest*

Of note, the outcome probabilities conditional on treatment $p_{i, 0}$ and $p_{i,1}$ probabilities of the causal forest are obtained by

$$p_{i,0}=E\left[ Y | X,W=0 \right]=\hat{m}\left( X \right)-\hat{e}\left( X \right)\hat{\tau}(X)$$

$$p_{i,1}=E\left[ Y | X, W=1 \right]=\hat{m}\left( X \right)+\left( 1-\hat{e}\left( X \right) \right)\hat{\tau}\left( X \right),$$

with $\hat{m}\left( X \right)=E[Y|X=x]$ and $\hat{e}\left( X \right)=E\left[ W | X=x \right]$ indicate the outcomes of two random forests, and $\hat{\tau}\left( X \right)=E\left[ Y_{i}\left( 0 \right)-Y_{i}\left( 1 \right) | X=x \right]$ indicates the treatment effect outcomes.
