# Supplementary material for: Performance metrics for models designed to predict treatment effect
Source: BMC Med Res Methodol. 2023 Jul 8;23:165. doi: 10.1186/s12874-023-01974-w (PMC10329397; doi:10.1186/s12874-023-01974-w)
Supplement: Supplementary file 3 — Additional file 3. Characteristics of patients in the Diabetes Prevention Program receiving lifestyle intervention, metformin, or placebo treatment. [file 12874_2023_1974_MOESM3_ESM.docx]

# **Additional file 3. Characteristics of patients in the Diabetes Prevention Program receiving lifestyle intervention, metformin, or placebo treatment.**

|  | Total | | |  | Lifestyle | |  | Metformin | |  | Placebo | |
| --- | --- | --- | --- | --- | --- | --- | --- | --- | --- | --- | --- | --- |
|  | N | % | Missing |  | N | % |  | N | % |  | N | % |
| Sample size | 3081 |  |  |  | 1024 |  |  | 1027 |  |  | 1030 |  |
| Diabetes | 655 | 21.3 |  |  | 148 | 4.8 |  | 215 | 7.0 |  | 292 | 9.5 |
| Female | 2053 | 66.6 |  |  | 685 | 22.2 |  | 669 | 21.7 |  | 699 | 22.7 |
| Ethnicity |  |  |  |  |  |  |  |  |  |  |  |  |
| Black | 644 | 20.9 |  |  | 204 | 6.6 |  | 221 | 7.2 |  | 219 | 7.1 |
| Hispanic | 508 | 16.5 |  |  | 178 | 5.8 |  | 162 | 5.3 |  | 168 | 5.5 |
| History of high blood glucose | 614 | 19.9 |  |  | 206 | 6.7 |  | 192 | 6.2 |  | 216 | 7.0 |
| Family history of diabetes | 2127 | 69.0 | 2 |  | 713 | 23.1 |  | 699 | 22.7 |  | 715 | 23.2 |
| Smoking | 216 | 7.0 |  |  | 67 | 2.2 |  | 69 | 2.2 |  | 80 | 2.6 |
| Hypertension | 835 | 27.1 |  |  | 286 | 9.3 |  | 267 | 8.7 |  | 282 | 9.2 |
| Gestational diabetes mellitus | 321 | 10.4 | 1 |  | 108 | 3.5 |  | 106 | 3.4 |  | 107 | 3.5 |
| Age (median, IQR) | 52 | [42; 57] |  |  | 47 | [42; 57] |  | 52 | [42; 57] |  | 47 | [42; 57] |
| BMI | 33 | [29; 37] |  |  | 33 | [29; 37] |  | 33 | [29; 37] |  | 33 | [29; 37] |
| Triglycerides | 141 | [99; 201] | 5 |  | 138 | [97; 200] |  | 137 | [98; 195] |  | 147 | [104; 207.5] |
| Haemoglobin $\boldsymbol{A}_{\boldsymbol{1}\boldsymbol{c}}$ | 5.9 | [5.6; 6.2] | 8 |  | 5.9 | [5.6; 6.2] |  | 5.9 | [5.6; 6.2] |  | 5.9 | [5.6; 6.2] |
| Fasting blood sugar | 105 | [101; 112] |  |  | 105 | [101; 112] |  | 105 | [100; 112] |  | 106 | [101; 112] |
