# Supplementary material for: Performance metrics for models designed to predict treatment effect
Source: BMC Med Res Methodol. 2023 Jul 8;23:165. doi: 10.1186/s12874-023-01974-w (PMC10329397; doi:10.1186/s12874-023-01974-w)
Supplement: Supplementary file 4 — Additional file 4. Parameter settings of Ridge regression and causal forest. [file 12874_2023_1974_MOESM4_ESM.docx]

**Additional file 4. Parameter settings**.

In the optimal model of the simulation study, we used a penalty of 0.0169 for the model including lifestyle intervention and 0.0189 for the model including metformin treatment for the Ridge regression. In the case study, the effect model had a penalty of 0.0281 when including lifestyle intervention and 0.0302 when including metformin treatment.

The causal forest contains several parameters that need to be tuned. We used 1000 trees to tune the following parameters: (1) sample fraction, the fraction of data used to build each tree, default is 0.5; (2) mtry, number of variables tried for each split, default is $\sqrt{p}+20$ with $p$ the number of variables; (3) minimal node size, minimum number of observations in each tree leaf, default is 5; (4) honesty fraction, the fraction used for determining splits, default is 0.5; (5) whether to honesty prune leaves such that no leaves are empty; (6) alpha, the maximum imbalance of a split, default is 0.05; and (7) imbalance penalty, penalizes imbalanced splits, default is 0[1]. The final parameters after tuning that were used to obtain treatment effect predictions with the causal forest are displayed in the Table below. To construct the final causal forest, we used 2000 trees.

**Tuned parameters of the causal forest.**

|  | Treatment assignment | |
| --- | --- | --- |
|  | Lifestyle | Metformin |
| Sample fraction | 0.372 | 0.400 |
| Mtry | 11 | 11 |
| Minimal node size | 1 | 3 |
| Honesty fraction | 0.772 | 0.676 |
| Honesty prune leaves | TRUE | TRUE |
| Alpha | 0.125 | 0.050 |
| Imbalance penalty | 0.029 | 0.031 |

**Reference**

1. Athey S, Tibshirani J, Wager S: **Generalized random forests**. *The Annals of Statisitics* 2019, **47**(2):1148-1178.
