# Supplementary material for: Performance metrics for models designed to predict treatment effect
Source: BMC Med Res Methodol. 2023 Jul 8;23:165. doi: 10.1186/s12874-023-01974-w (PMC10329397; doi:10.1186/s12874-023-01974-w)

**Additional file 5. The probability of the potential outcome of diabetes under lifestyle intervention versus under control treatment.** This Figure displays the predicted probabilities of the potential treatment under the two treatment alternatives, where the distance to the diagonal line is the treatment effect. The Figures displays the “optimal model” in panel **A**, and three “perturbed models” that overestimate average treatment effect (panel **B**), risk heterogeneity (panel **C**), and treatment effect heterogeneity (panel **D**).


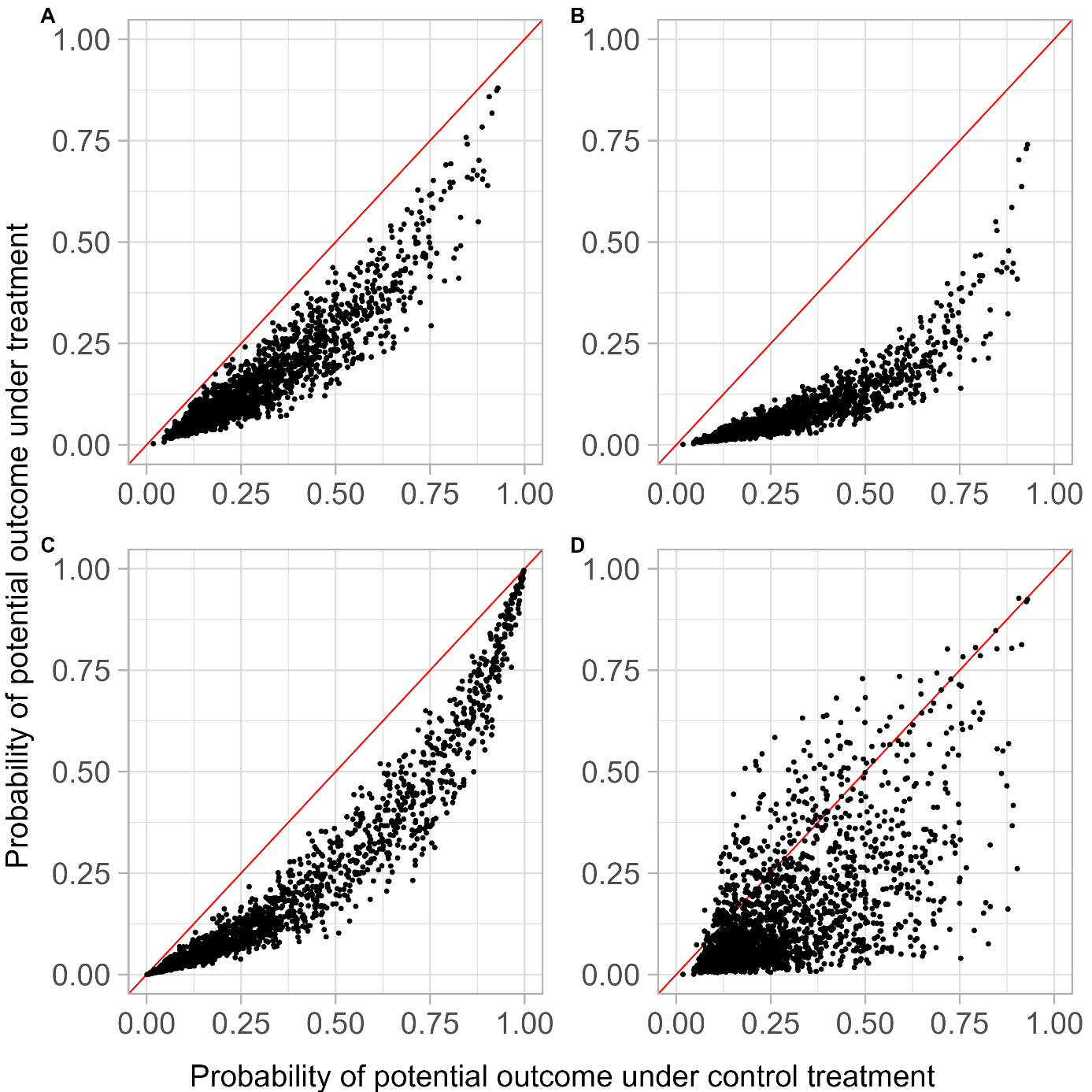

Supplement: Supplementary file 5 — Additional file 5. The probability of the potential outcome of diabetes under lifestyle intervention versus under control treatment predicted by the “optimal model”, and three “perturbed models” that overestimate average treatment effect, risk heterogeneity, and treatment effect heterogeneity. [file 12874_2023_1974_MOESM5_ESM.docx]
