# Supplementary material for: Performance metrics for models designed to predict treatment effect
Source: BMC Med Res Methodol. 2023 Jul 8;23:165. doi: 10.1186/s12874-023-01974-w (PMC10329397; doi:10.1186/s12874-023-01974-w)
Supplement: Supplementary file 7 — Additional file 7. Calibration plots of pairwise treatment effect of simulated data from patients receiving lifestyle intervention or metformin. This Figure depicts observed versus predicted pairwise treatment effect by smoothed calibration curves (blue line) and quantiles of predicted pairwise treatment effect (black dots) of simulated data from the lifestyle intervention or metformin versus placebo treatment. Observed pairwise treatment effect was obtained by matching patients based on patient characteristics. Smoothed calibration curves were obtained by local regression of the observed pairwise treatment effect of matched patient pairs on predicted pairwise treatment effect of matched patient pairs. For prediction of individualized treatment effect, we used the “perturbed model” that underestimates average treatment effect for lifestyle intervention (panel A) and metformin treatment (panel B). The average treatment effect is 7.1 and 3.5, respectively. [file 12874_2023_1974_MOESM7_ESM.docx]

**Additional file 7. Calibration plots of pairwise treatment effect of simulated data from patients receiving lifestyle intervention or metformin.** This Figure depicts observed versus predicted pairwise treatment effect by smoothed calibration curves (blue line) and quantiles of predicted pairwise treatment effect (black dots) of simulated data from the lifestyle intervention or metformin versus placebo treatment. Observed pairwise treatment effect was obtained by matching patients based on patient characteristics. Smoothed calibration curves were obtained by local regression of the observed pairwise treatment effect of matched patient pairs on predicted pairwise treatment effect of matched patient pairs. For prediction of individualized treatment effect, we used the “perturbed model” that underestimates average treatment effect for lifestyle intervention (panel **A**) and metformin treatment (panel **B**). The average treatment effect is 7.1 and 3.5, respectively.

| **A** | **B** |
| --- | --- |
| 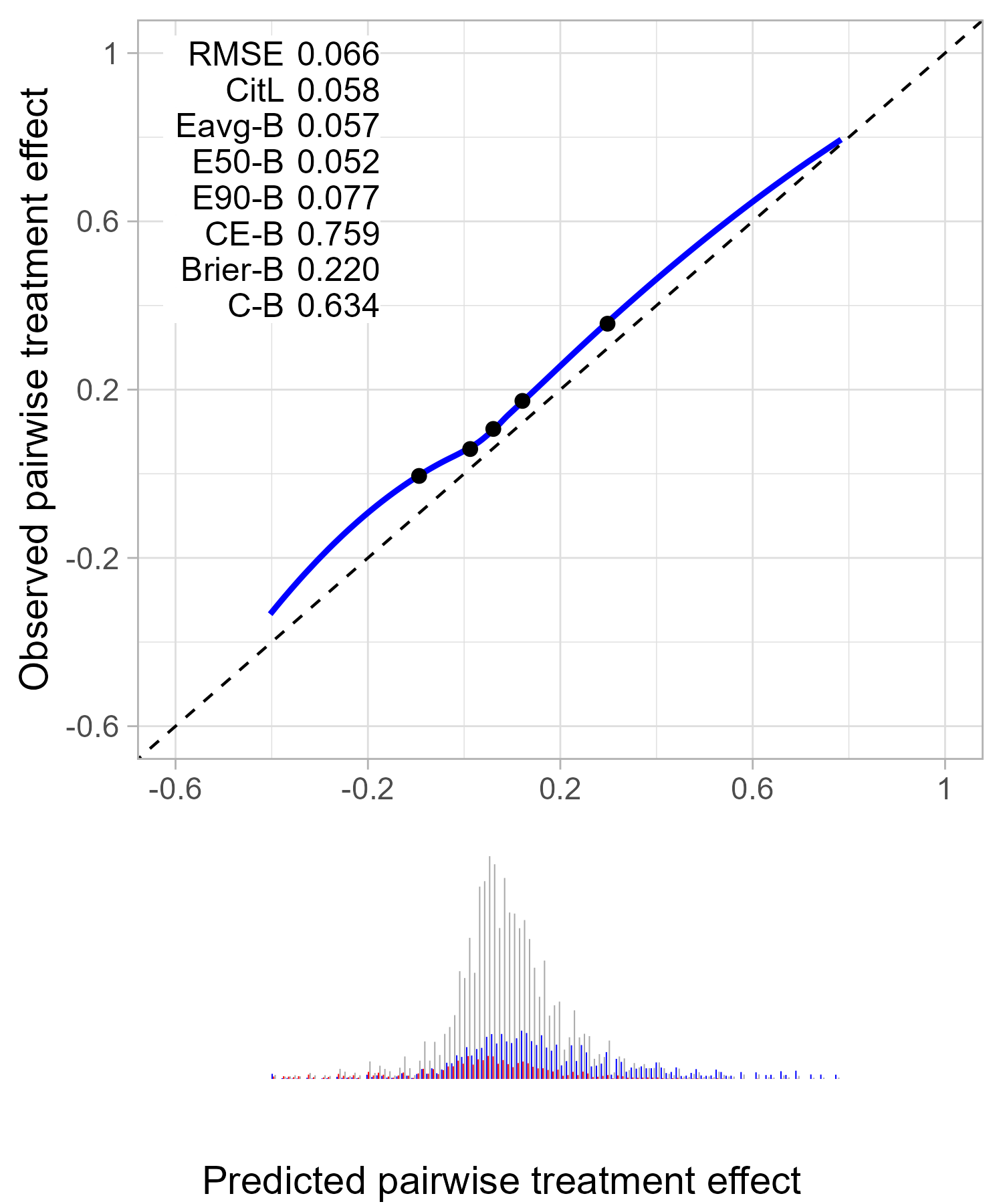 | 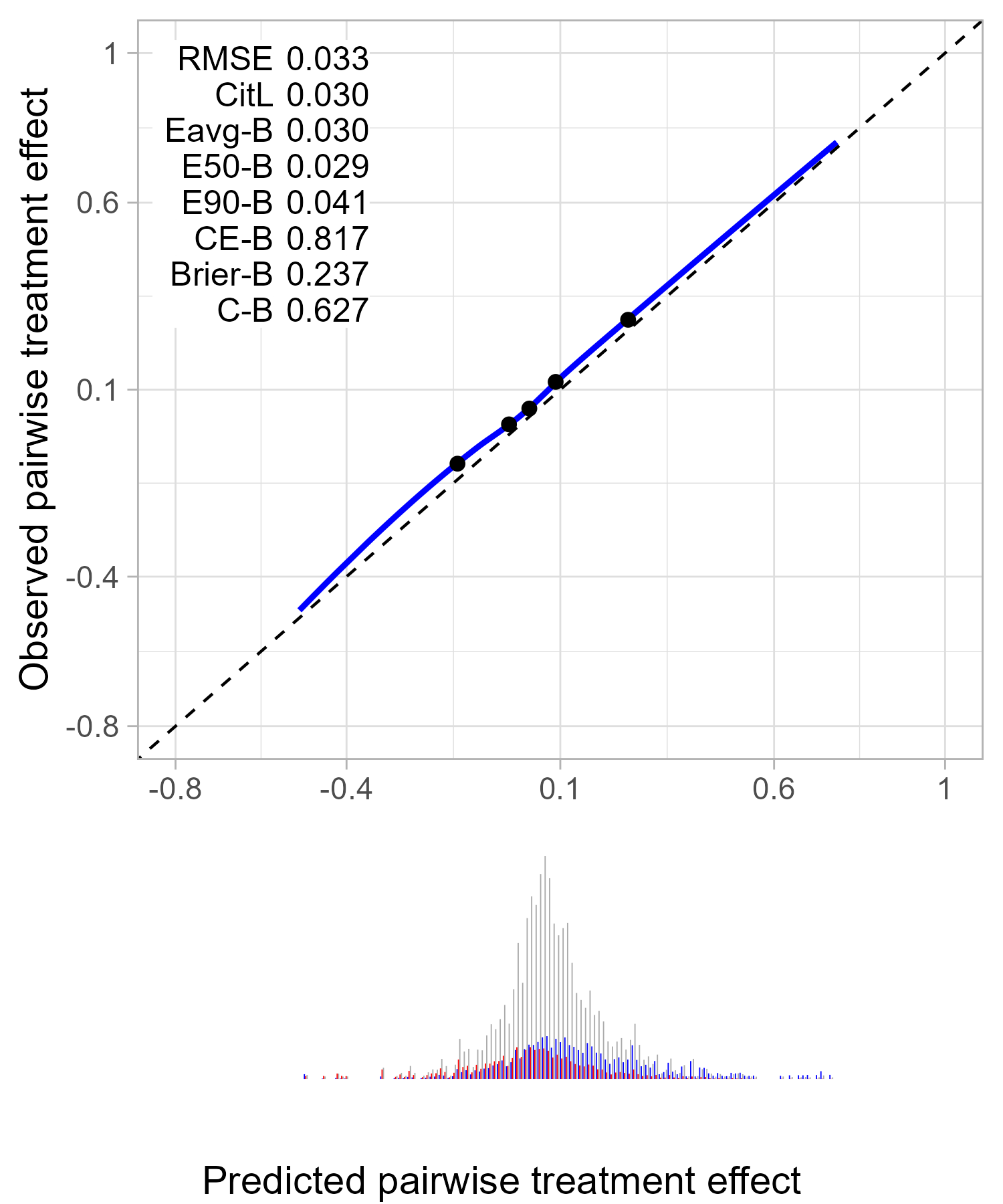 |
