# Supplementary material for: Performance metrics for models designed to predict treatment effect
Source: BMC Med Res Methodol. 2023 Jul 8;23:165. doi: 10.1186/s12874-023-01974-w (PMC10329397; doi:10.1186/s12874-023-01974-w)
Supplement: Supplementary file 8 — Additional file 8. Calibration plots of pairwise treatment effect of simulated data from patients receiving metformin intervention. This Figure depicts observed versus predicted pairwise treatment effect by smoothed calibration curves (blue line) and quantiles of predicted pairwise treatment effect (black dots) of simulated data from the metformin versus placebo treatment. Observed pairwise treatment effect was obtained by matching patients based on patient characteristics. Smoothed calibration curves were obtained by local regression of the observed pairwise treatment effect of matched patient pairs on predicted pairwise treatment effect of matched patient pairs. For prediction of individualized treatment effect, we used a treatment effect modelling approach for the “optimal model” (panel A) and three “perturbed models” that overestimate average treatment effect (panel B), risk heterogeneity (panel C), and treatment effect heterogeneity (panel D). The average treatment effect is 6.6, 11.9, 6.6 (after a correction of with -0.085), and 6.6 (after a correction of with -0.16), respectively. [file 12874_2023_1974_MOESM8_ESM.docx]

**Additional file 8. Calibration plots of pairwise treatment effect of simulated data from patients receiving metformin intervention.** This Figure depicts observed versus predicted pairwise treatment effect by smoothed calibration curves (blue line) and quantiles of predicted pairwise treatment effect (black dots) of simulated data from the metformin versus placebo treatment. Observed pairwise treatment effect was obtained by matching patients based on patient characteristics. Smoothed calibration curves were obtained by local regression of the observed pairwise treatment effect of matched patient pairs on predicted pairwise treatment effect of matched patient pairs. For prediction of individualized treatment effect, we used a treatment effect modelling approach for the “optimal model” (panel **A**) and three “perturbed models” that overestimate average treatment effect (panel **B**), risk heterogeneity (panel **C**), and treatment effect heterogeneity (panel **D**). The average treatment effect is 6.6, 11.9, 6.6 (after a correction of $\beta_{W}$ with -0.085), and 6.6 (after a correction of $\beta_{W}$ with -0.16), respectively.

**
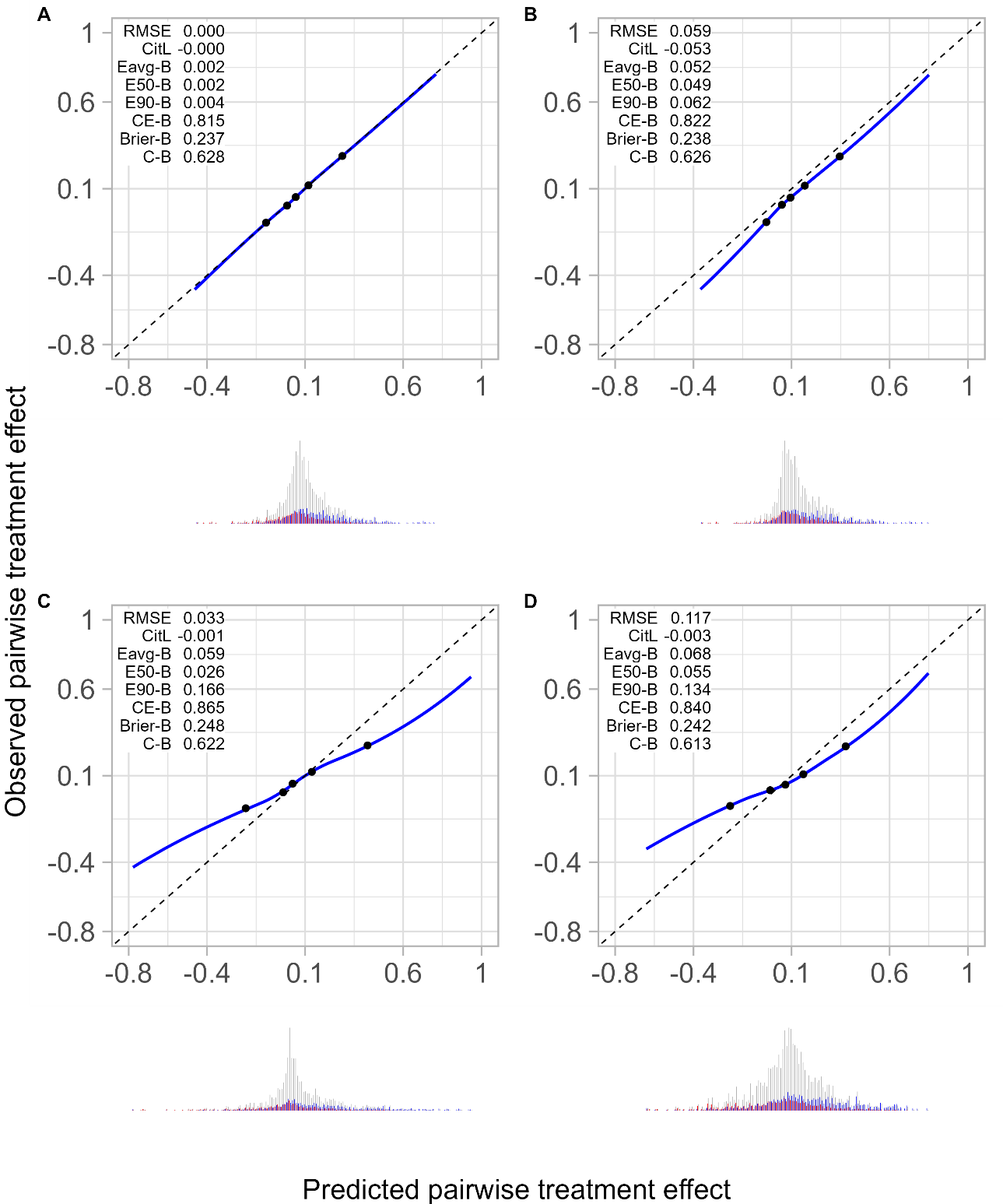
**
