# Supplementary material for: Performance metrics for models designed to predict treatment effect
Source: BMC Med Res Methodol. 2023 Jul 8;23:165. doi: 10.1186/s12874-023-01974-w (PMC10329397; doi:10.1186/s12874-023-01974-w)

**Additional file 9. Calibration plot of pairwise treatment effect of training and test data of lifestyle intervention.** This Figure depicts observed versus predicted pairwise treatment effect by smoothed calibration curves (blue line with 95% confidence interval displayed by grey shaded area) and quarters of predicted pairwise treatment effect (black dots) of lifestyle intervention versus placebo treatment. Observed pairwise treatment effect was obtained by matching patients based on patient characteristics. Smoothed calibration curves were obtained by local regression of the observed pairwise treatment effect of matched patient pairs on predicted pairwise treatment effect of matched patient pairs. For prediction of treatment effect, we used: a risk modelling approach (panel **A**; **B**), a treatment effect modelling approach (panel **C**; **D**), and a causal forest (panel **E**; **F**). The models are trained on 70 percent of the data (panel **A**; **C**; **E**) and evaluated on the other 30 percent of the data (**B**; **D**; **F**). Confidence intervals around the metric values were obtained using 100 bootstrap samples.


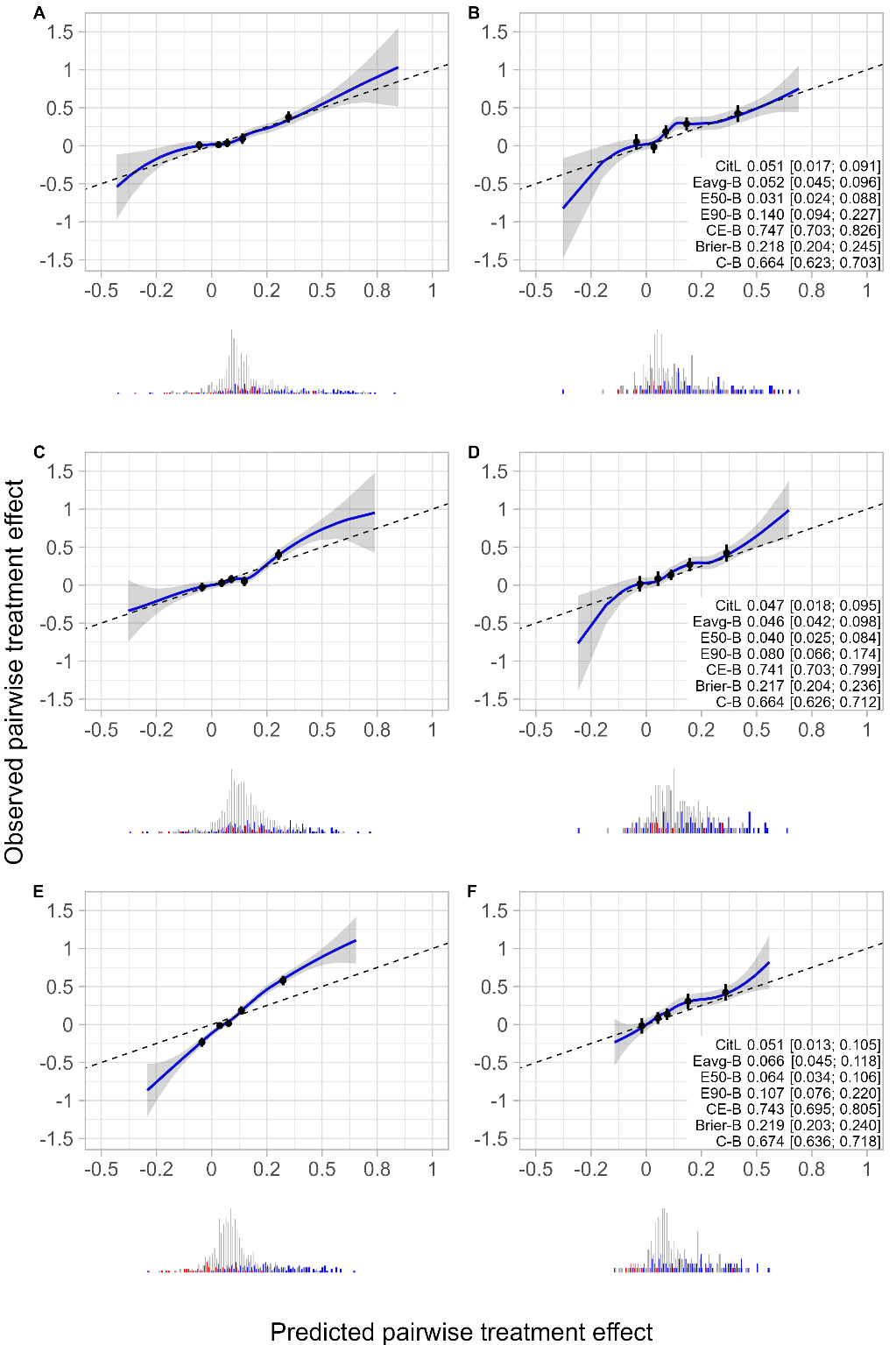

Supplement: Supplementary file 9 — Additional file 9. Calibration plot of pairwise treatment effect of training and test data of lifestyle intervention. This Figure depicts observed versus predicted pairwise treatment effect by smoothed calibration curves (blue line with 95% confidence interval displayed by grey shaded area) and quarters of predicted pairwise treatment effect (black dots) of lifestyle intervention versus placebo treatment. Observed pairwise treatment effect was obtained by matching patients based on patient characteristics. Smoothed calibration curves were obtained by local regression of the observed pairwise treatment effect of matched patient pairs on predicted pairwise treatment effect of matched patient pairs. For prediction of treatment effect, we used: a risk modelling approach (panel A; B), a treatment effect modelling approach (panel C; D), and a causal forest (panel E; F). The models are trained on 70 percent of the data (panel A; C; E) and evaluated on the other 30 percent of the data (B; D; F). Confidence intervals around the metric values were obtained using 100 bootstrap samples. [file 12874_2023_1974_MOESM9_ESM.docx]
